# Malignant Peripheral Nerve Sheath Tumour: CT and MRI Findings

**DOI:** 10.1155/2013/517879

**Published:** 2013-07-18

**Authors:** Massimiliano Sperandio, Isabelle Di Poce, Aurora Ricci, Roberta Di Trapano, Elisa Costanzo, Pierfrancesco Di Cello, Fabio Pelle, Luciano Izzo, Giovanni Simonetti

**Affiliations:** ^1^Department of Diagnostic Imaging, Molecular Imaging, Interventional Radiology and Radiation Therapy, University Hospital Tor Vergata, Viale Oxford, 81-00133 Rome, Italy; ^2^Department of Surgery “P. Valdoni”, Policlinico Umberto I, Sapienza University, Viale del Policlinico, 155-00161 Rome, Italy

## Abstract

Malignant peripheral nerve sheath tumour (MPNST) is extremely rare malignancy in the general population, occurring more frequently in patients with Neurofibromatosis type 1 (NF1). In the literature five cases of MPNST arising from the parapharyngeal space (PPS) in patients without neurofibromatosis have been reported. We report imaging techniques in a patient with MPNST in the PPS, who had neither a family history nor sign of NF1. Computed tomography (CT) scan and magnetic resonance imaging (MRI) were performed for a correct therapeutic planning. CT and MRI findings were correlated with hystopathological diagnosis.

## 1. Introduction

Malignant peripheral nerve sheath tumour (MPNST) is an extremely rare malignancy. It usually encountered in patients with Neurofibromatosis type 1 (NF1) with an incidence of 2–5%, while in the general population it has an incidence of 0,001% [1, 2]. It arises from the Schwann cells of peripheral nerves, and it rarely involves the cranial nerves [3–5]. We report a case of malignant peripheral nerve sheath tumour in the parapharyngeal space (PPS) arising in patient who had neither a family history nor sign of NF1.

## 2. Case Presentation

A 71-year-old man was admitted to our observation with a five months history of a gradually enlarging pulsanting painful neck mass, localized in the left laterocervical side. He reported dysphagia and breathing difficulty since about four weeks. He did not report symptoms associated with cranial nerve deficit. 

Intraoral inspection demonstrated a swelling in the tonsillar region without erosion of the mucosa. 

Computed tomography (CT) scan, performed with a 64-row scanner (LightSpeed VCT, General Electric Medical System, Milwaukee, WI, USA), showed a well-defined isodense expansive lesion in the left PPS, which determined bulging of the oropharyngeal wall. 

**Figure 1:**
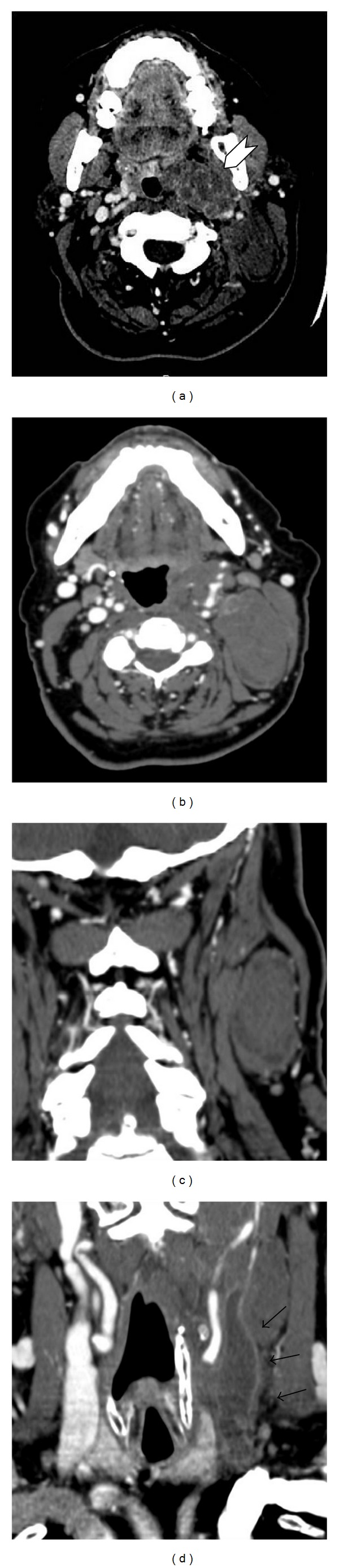
CT scan shows an expansive lesion in the left PPS. (a-b) Axial CT scan after injection of contrast medium shows an inhomogeneous contrast enhancement of the mass in the left PPS; (c) coronal-MPR shows the extension of the lesion in the ipsilateral laterocervical space; (d) the mass compress the ipsilateral the left internal jugular vein with an evident cleavage plane; the vein presents an extensive thrombosis.

After the injection of contrast medium organo-iodized (Iomeron 350 mg/mL, Bracco Imaging, Milan, Italy), the mass showed inhomogeneous contrast enhancement (Figure 1) because of the presence of low-density foci corresponding to necrosis, suggestive of malignant lesion. The lesion displaced the ipsilateral internal carotid artery and the left internal jugular vein without evidence of invasion; the latter presented an expansive thrombosis extending distally up to the confluence with anonymous trunk (Figure 1). 

Different diagnostic hypotheses were formulated: pleomorphic adenoma, neurogenic tumour, and lymphnode pathology. A subsequent dynamic contrast-enhanced MRI with a 1.5 T unit (Gyroscan Intera, Philips Medical Systems, Best, The Netherlands) was performed with the head-neck coil to better characterize the mass and to assess the topographical relationship of the tumour with vessels and with neighbouring structures. MRI protocol included axial and coronal T2 (T2WI), axial and coronal fat-saturated T2, axial e coronal T1 (T1WI), and axial T1W fat-saturated postcontrast agent (gadopentetic acid and dimeglumine salt, Magnevist; Schering, Berlin, Germany).

MRI confirmed the presence of a well-defined rounded lesion localized in the left PPS. The lesion compressed the pharynx, and it had a cranial extension almost up to the Eustachian tube. 

The mass presented a signal hyperintensity in T2WI and in T2WI fat-saturated sequences and a signal hypointensity in T1WI sequences.

**Figure 2:**
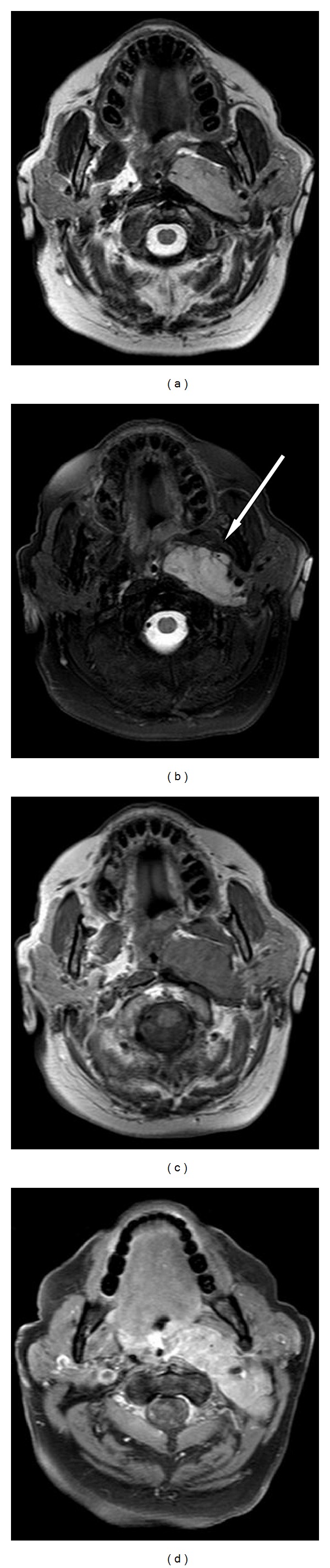
MRI evaluation. (a-b) Axial T2-weighted MRI image (TSE, 4000/80 [TR/TE]) and T2-weighted fat saturated (SPIR 3640/70/200 [TR/TE/TI]) show a well-defined hyperintense mass which determinates bulging of the pharyngeal wall; (c) axial T1-weighted image (265/7.5 [TR/TE]) showed an isointense mass compared to the muscle signal intensity; (d) axial T1-weighted image after gadolinium injection evidences an intense and inhomogeneous contrast enhancement of the mass.

Post-Gadolinium T1WI fat-saturated sequences showed an early inhomogeneous contrast enhancement of the lesion (Figure 2). The multiplanar capability of MRI demonstrated ipsilateral vascular structures dislocation and a low signal intensity after contrast medium administration localized in the left internal jugular vein, suggestive for an extensive thrombus. It also revealed the relationship between the tumour and the ipsilateral sternocleidomastoid muscle, which appeared compressed but not infiltrated.

The fat tissue thickness appeared reduced. Multiple increased in size lymph nodes were detected behind the left mandibular angle and in the bilateral laterocervical spaces.

**Figure 3:**
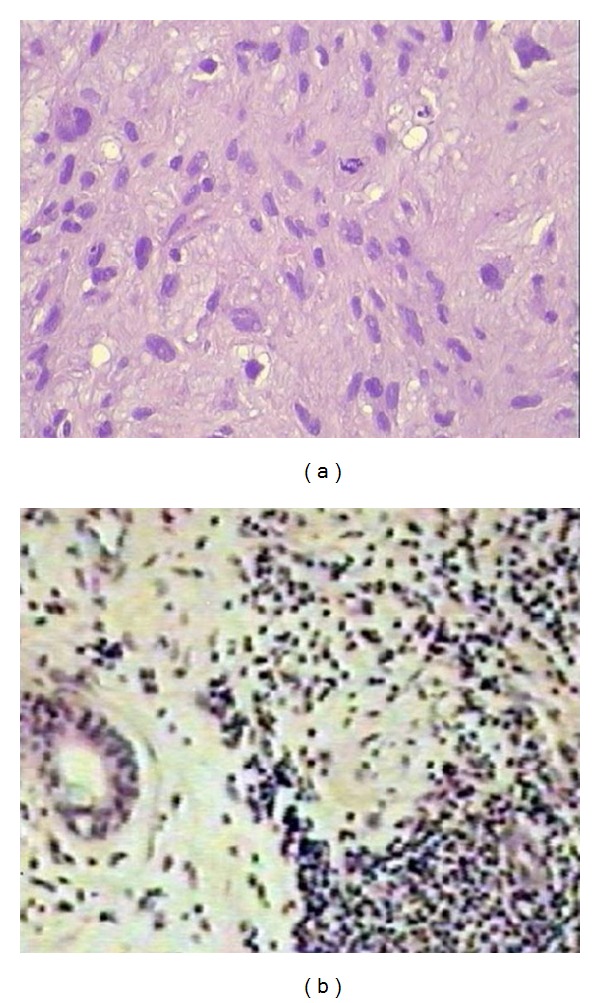
Photomicrograph of the histology. (a) The mass is made up of relatively hypo- and hypercellular areas. The cells are spindle or polyhedralshaped and show moderate degree of cellular pleomorphism and frequent mitoses (haematoxylin and eosin, original magnification ×100); (b) immunohistochemically the tumour cells stained strongly positive with monoclonal antibody vimentin (original magnification ×400).

The patient underwent an incisional biopsy with multiple Tru-Cut needle biopsy which revealed a neoplasm composed of fusiform cells with small necrotic foci. Immunohistochemistry demonstrated strongly positive staining for neurone specific enolase (NSE), slightly positive staining for a smooth, actine, and positive staining for S-100 protein, consistent with malignant neurosarcoma or MPNST (Figure 3). 

CT total body examination showed no evidence of metastases. 

The patient underwent a transcervical surgical excision of the tumour without any subsequent complication, followed by radiation therapy.

## 3. Discussion

MPNST is a rare sarcomas arising from the Schwann cells of peripheral or exceptionally from the cranial nerves [3–7]. It can occur de novo or more frequently within pre-existing plexiform neurofibromas; thus patients with neurofibromatosis and plexiform neurofibromas warrant increased surveillance for development of these tumours [8].

Thus only few of these tumors occur in the head and neck regions [9]. In these cases we reported a MPNST developed from the PPS.

In the literature five cases of MPNST arising from the PPS in patients without Neurofibromatosis have been reported [10, 11]. Tumours of PPS are uncommon and represent 0.5–1% of all head and neck neoplasms and are mostly benign; only 20–30% of these are malignant [9]. They can originate from structures within the space, but they can also be localization of metastatic disease [12, 13]. The most frequent benign tumor of PPS is represented by the pleomorphic adenoma, which arises from the salivary glands (40–50%); the mucoepidermoid carcinoma (MEC) is the most common malignant neoplasm [13]. Symptoms are usually subtle and late because of the deep location in the neck; PPS tumours become clinically detectable when their size achieves a diameter of 2-3 cm. The growing tumour determinates a specific symptoms, which include a visible parotid swelling, oropharyngeal swelling, dysphagia, dyspnoea, and nasal obstruction [14]. 

The spectrum of differential diagnosis of a neoplastic mass in the PPS commonly includes salivary gland tumour, neurogenic tumour, skull base, and vertebral tumour such as meningioma or chordoma, rhabdomyosarcoma, and more rarely, Castleman's disease, which arises from adjacent nodes and extends into the space [10].

The PPS is a complex anatomical area, and it is difficult to be examined; it is described as an inverted pyramid divided into two spaces: the prestyloid or “true parapharyngeal” space and the poststyloid or carotid or retrostyloid space. The first one includes fat, a small portion of the retromandibular parotid gland, minor or ectopic salivary glands, branches of the mandibular division of the trigeminal nerve, and some lymph nodes. The poststyloid space includes the internal carotid artery, the internal jugular vein, cranial nerves (IX–XII), the sympathetic chain, and lymph nodes [15].

CT and MRI images are fundamental to evaluate tumours of this anatomical area, while there is not a reliable sonographic window to allow an ultrasound examination. 

CT is useful to evaluate the PPS in but MRI results in the preferred technique for a better contrast resolution. While CT is useful to assess the tumour extension and eventual metastasis (the more frequent are bone and lung metastasis), MRI can reveal the nerve of origin, and it is more accurate to evaluate the topographical relationship of the tumour with neighbouring structures, especially vascular, muscular structures and fat planes involvement. In particular MRI distinguishes the lesion from the fat tissue better than CT, whose dislocation and thinning of the fat tissue thickness have a critical importance in localizing the space of origin of a neck lesion [15].

Histologically MPNST is a spindle-cell sarcoma. The common type of malignant schwannoma consists of rounded or fusiform cells arranged in sweeping fascicles and recapitulating the features of normal Schwann cells. 

Other hystological types can be encountered, such as glandular malignant schwannoma, malignant epithelioid schwannoma, or malignant Triton tumour, where both malignant schwannoma and rhabdomyosarcoma are associated. The rhabdomyoblastic type is more often encountered in the head and neck [16]. 

If some authors consider the fine needle aspiration biopsy useful diagnostically, the definitive diagnosis is immunohistological. In particular, histology can allow differential diagnosis between MPNST and other types of fusicellular sarcoma on the basis of immunohistochemical markers, including S-100 protein and NSE [17, 18].

Histopathological characterization can be performed by tru-cut biopsy, open incisional biopsy, or excisional biopsy. 

Although there is an established management protocol, surgical resection using different techniques (transoral, transcervical, transparotid transcervical, transcervical transmandibular, or infratemporal) is the mainstay of treatment of MPNST. Surgical treatment is usually followed by radiation therapy, with or without adjuvant chemotherapy [18, 19].

Surgical candidates include patients with primary malignant neoplasms for which surgery is appropriate therapy, patients with select metastatic malignancies (e.g., papillary thyroid carcinoma), benign salivary gland neoplasms, neurogenic neoplasms with existing cranial nerve deficits, and neoplasms with mass effect symptoms.

If the transcervical approach remains the most commonly used surgical approach to the parapharyngeal space, the transoral approach is now considered for selecting small and accessible neoplasms. Recently, transoral endoscopic and robotic approaches to the parapharyngeal space have been described [19].

## 4. Conclusion

MPNST is a very rare disease, especially in patients without neurofibromatosis and in the PPS. Neoplasms developed from the PPS represent a diagnostic issue because of the complexity of this anatomical area. Imaging tools are fundamental to characterize and localize the tumour and for a correct therapeutic planning. MRI represents the technique of choice, because it presents a better contrast resolution than CT scan.
